# Differences in patterns of sexual assault among female victims preceding and during the COVID-19 pandemic: an analysis of encounters in an emergency department

**DOI:** 10.1007/s12024-023-00725-z

**Published:** 2023-10-10

**Authors:** Caroline M. Klasen, Leandra Teltrop, Matthias H. Belau, Larissa Lohner, Benjamin Ondruschka, Kerstin Riecke, Susanne Reuter, Barbara Schmalfeldt, Sandra Wilmes, Isabell Witzel

**Affiliations:** 1 Department of Gynecology, University Medical Center Hamburg-Eppendorf, Martinistrasse 52, Hamburg, 20246 Germany; 2 Institute of Medical Biometry and Epidemiology, University Medical Center Hamburg-Eppendorf, Hamburg, Germany; 3 Institute for Forensic Medicine, University Medical Center Hamburg, Hamburg, Germany; 4 Department of Gynecology, University Hospital Zurich, University of Zurich, Zurich, Switzerland

**Keywords:** Sexual assaults, COVID-19 pandemic, Lockdown, Genital injury, Reporting

## Abstract

The aim of this study was to evaluate how the COVID-19 pandemic may have impacted the number and patterns of sexual assault victims within a German metropolitan city. A retrospective single center analysis of the gynecology examination reports of all women presenting to the emergency department of a university hospital after a sexual offense between 03/2013 and 02/2021 (n = 1167). Comparison of the first year of the pandemic 03/2000-03/2021) to previous years (03/2017-02/2020) and comparison of periods of government-imposed social distancing (03/12/2020-05/23/2020 and 10/23/2020-02/28/2021) with corresponding periods of pre-pandemic years. The overall number of sexual assault cases did not change during the first year of the COVID-19 pandemic. However, during the stay-at-home orders, the number of women presenting to the emergency department decreased by 38% (n=45 vs. 72). Fewer victims filed a police report during the pandemic (49.5% vs. 73.9%, p<0.001) and the lockdown period (50% vs. 76.5%, p<0.001). Less genital injuries after sexual assault were detected during the pandemic (14.3% vs. 25.2%, p<0.02), but there was an increase of illegal substance abuse (19.5% vs. 9.3%, p<0.003). During the stay-at-home orders fewer victims reported alcohol consumption (42.4% vs. 62.5 %, p<0.023). Despite the decrease in sexual offense related police reports, the number of sexual assault cases remained consistent, and the usage of illegal drugs increased during the COVID-19 pandemic. These findings represent the importance of providing support to sexual assault victims, as well as the implementation of preventative measures, especially in times of crisis.

## Background

Sexual assault is a serious public health problem affecting up to a third of women worldwide [1]. In Germany, 29.836 cases of sexual assault and rape were reported to the police in 2021; cases with female victims making up 92.2% of those reports [2]. However, it is assumed that the actual number of cases is much higher; a recent dark-field study revealed that only 9,5% of sexual assault victims filed a police report [3]. Psychological factors, such as anxiety, shame, fear, or negative perceptions of the police, as well as pre-existing relationships to the assailants, sometimes keep victims from reporting sexual assault cases to the police [4, 5]. Previous studies that evaluated numbers and characteristics of sexual violence victims indicate that, regardless of police reporting, post-sexual assault care is mainly provided by gynecologists or specially trained nurses at the emergency departments of hospitals [6–8]. Recent studies from large metropolitan cities in Germany, indicated that the mean age of the female sexual assault victims is 26 years and in about half of the cases, the reported suspect is not a stranger to the victim. In more than half of cases, the assault happens in temporal connection with voluntary alcohol consumption. Extragenital injury is more frequent in two thirds of cases and genital injury is reported in one third of cases [8, 9].

On January 27^th^, 2020, the first COVID-19 case in Germany was confirmed. With the spread of the infection, preventive measures were replaced by containment measures; including a country-wide lockdown from March 2020 until May 2020, with contact restrictions and stay-at-home orders. A second lockdown followed from October 2021 until March 2021.

Historically, in times of crisis, natural disasters, and previous epidemics, cases of violence against women have risen [10–13]. International research is beginning to suggest an increase in domestic sexual violence during the COVID-19 pandemic [14, 15] and a decrease in emergency room admissions and access to health care services [16, 17]. However, to our knowledge, publications regarding the effects of the COVID-19 pandemic on the reporting of women after sexual assaults to the health care system or the police are missing to date.

Thus, we aimed to compare patterns of sexual assault among victims during the COVID-19 pandemic in an emergency department of the University Medical Center in Hamburg, Germany. We hypothesized that emergency department encounters differed in sexual assault patterns during the COVID-19 pandemic compared with those before the pandemic.

## Methods

### Study design, sample, and procedure

We assessed the data of all female emergency department encounters at the University Medical Center Hamburg-Eppendorf between the 1^st^ of January 2013 and the 30^th^ of April 2021, in which a sexual assault was reported. Routine examinations of female sexual assault survivors included gynecological and forensic examinations by specially trained physicians. This involved classification of the sites of injury (genital, extragenital), the site of penetration (vaginal, oral, anal), and the type of penetration (penile, oral, with fingers or hand, with an object). Information on genital injuries was categorized as follows: none, external genitals, internal genitals, anal, and/or multiple genital injuries. Extragenital injuries were categorized as follows: none, head, neck, trunk, upper limb, lower limb, and multiple extragenital injuries. In addition, sexual assault survivors were asked about their relationship to the perpetrator (stranger, acquaintance), whether this was their first sexual assault (no, yes), whether they reported the incident to the police (no, yes), and where the crime happened (victim's home, perpetrator's home, nightclub/bar, other). Information on voluntary and involuntary substance use (alcohol, illicit drugs) was categorized (no/yes).

**Fig. 1:**
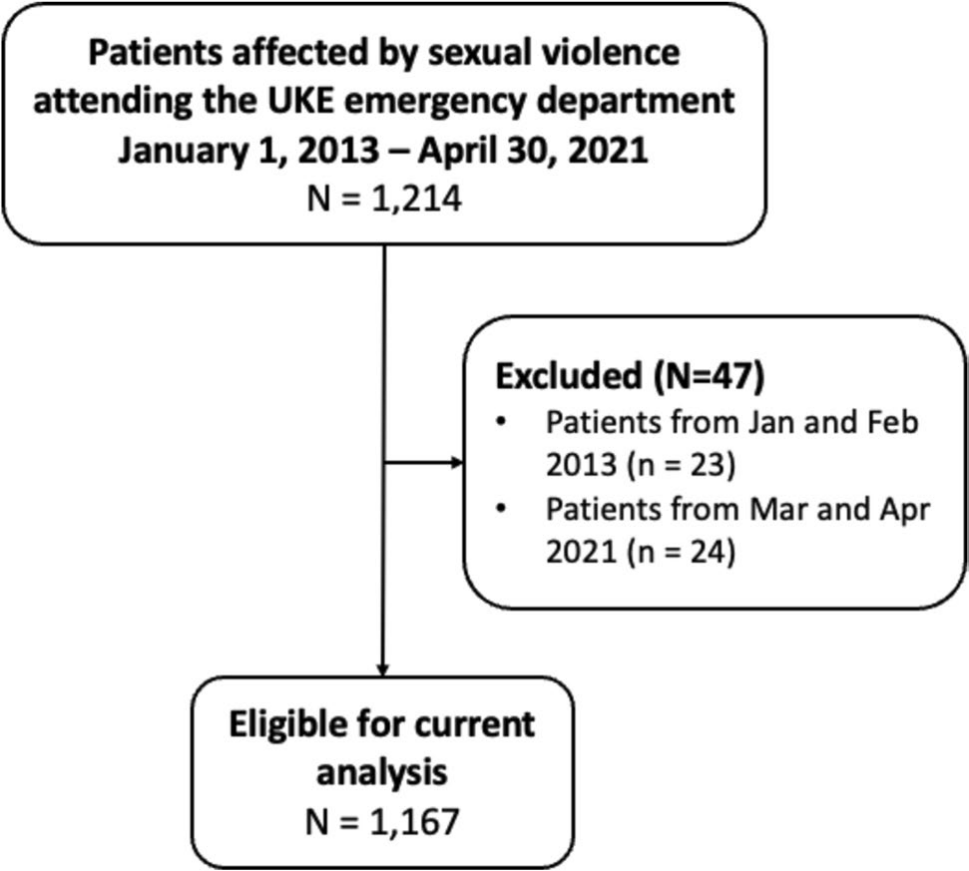
Patient flow chart

A total of 1,214 women reported a sexual assault to the emergency department between Jan 2013 and April 2021 (see Fig. 1). The pandemic was declared by the World Health Organization on the 11^th^ of March 2020 [18], however, we used March 1^st^, 2020 as the cut-off date for group comparison preceding and during the pandemic. Accordingly, data are available for one year during the pandemic (March 2020 to February 2021), and for seven years preceding the pandemic (March 2013 to February 2020). Given that female sexual assault cases might have been influenced by public health recommendations or government regulations, we further compared periods of government-imposed social distancing during the pandemic (March 12^th^ to May 23^rd^, 2020, and October 23^rd^ to February 28^th^, 2021) with the corresponding periods of pre-pandemic years.

## Statistical analyses

Statistical analyses were performed using STATA MP, version 17. Descriptive statistics were used to examine the patient’s characteristics. Differences in patterns of sexual assault among women were analyzed using the chi-squared test, Fisher’s exact, and t-test (p<0.05).

## Results

### Pandemic and number of victims

Prior to the pandemic (March 2013 to February 2020), on average 148 women visited the Hamburg-Eppendorf emergency department for sexual assault each year. During the pandemic (March 2020 to February 2021), 130 women visited these facilities. Table 1 presents the characteristics of the participants by period during the pandemic (03/202-02/2021) and pre-pandemic (03/2013-02/2020).

**Table 1:** Patient characteristics by period (N=1,167)

	**During pandemic** **03/2020 – 02/2021** n=130		**Pre-pandemic** **03/2013 – 02/2020** n=1,037		***p*-value**
	**n**	**%**	**n**	**%**	
**Age**					0.994
Mean (SD)	26.6 (10.6)		26.6 (12.0)		
* Missing values*	*2*		*9*		
**Victim-offender relationship**					0.113
Stranger	40	37.7	428	45.8	
Acquaintance	66	62.3	506	54.2	
* Missing values*	*24*		*103*		
**First sexual assault**					0.590
No	9	7.5	88	9.0	
Yes	111	92.5	892	91.0	
* Missing values*	*10*		*57*		
**Place of sexual assault**					0.287
Apartment of the victim	29	26.4	82	25.6	
Apartment of the offender	40	36.3	100	31.3	
Nightclub, bar	12	10.9	60	18.7	
Other	29	26.4	78	24.4	
* Missing values*	*20*		*716*		
**Reported to police**					<0.001
No	55	50.5	212	26.1	
Yes	54	49.5	599	73.9	
* Missing values*	*21*		*226*		
**Genital injury**					0.020
None	96	85.7	702	74.8	
External genitals	13	11.6	118	12.6	
Internal genitals	2	1.8	47	5.0	
Anal	1	0.9	29	3.1	
Multiple genital injuries	0	0.0	42	4.5	
* Missing values*					
**Extragenital injury**					0.871
None	18	23.4	208	23.3	
Head, neck, or trunk	5	6.5	63	7.0	
Upper limb	3	3.9	46	5.2	
Lower limb	12	15.6	104	11.6	
Multiple extragenital injuries	39	50.6	472	52.9	
* Missing values*	*53*		*143*		
**Alcohol use**					0.816
No	36	40.0	353	38.8	
Yes	54	60.0	558	61.2	
* Missing values*	*40*		*126*		
**Drug use**					0.003
No	66	80.5	800	90.7	
Yes	16	19.5	82	9.3	
* Missing values*	*48*		*155*		

*n* quantity, *%* percentage, *SD* standard deviation

### Lockdown during the pandemic and number of victims

During the two periods of government-imposed social distancing (03/12/2020-05/11/2020 and 10/23/2020-03/01/2020), fewer women (n=45) presented to the emergency department after a claimed sexual assault, compared to 72 women on average in the corresponding periods of the previous years (2013-2019).

Table 2 shows differences between government-imposed social distancing and the corresponding period of pre-pandemic years across patient characteristics.

**Table 2:** Government-imposed social distancing and corresponding period of previous non-COVID-19 years across patient characteristics (N=552)

	**Periods**^**a**^** of government-imposed social distancing during pandemic** **2020 – 2021** n=45		**Corresponding periods**^**#**^** of the pre-pandemic years** **2013 – 2020** n=507		***p*-valu**e
	**n**	**%**	**n**	**%**	
**Age**					0.476
Mean (SD)	27.9 (11.3)		26.6 (11.4)		
* Missing values*	*1*		*4*		
**Victim-offender relationship**					0.065
Stranger	10	27.8	196	43.6	
Acquaintance	26	72.2	254	56.4	
* Missing values*	*9*		*57*		
**First sexual assault**					0.488
No	38	92.7	422	89.2	
Yes	3	7.3	51	10.8	
* Missing values*	*4*		*34*		
**Place of sexual assault**					0.372
Apartment of the victim	11	28.9	49	27.8	
Apartment of the offender	14	36.8	58	33.0	
Nightclub, bar	3	7.9	34	19.3	
Other	10	26.3	35	19.9	
* Missing values*	*7*		*331*		
**Reported to police**					<0.001
No	19	50.0	95	23.5	
Yes	19	50.0	309	76.5	
* Missing values*	*7*		*103*		
**Genital injury**					0.319
None	35	89.7	340	75.9	
External genitals	4	10.3	50	11.2	
Internal genitals	0	0.0	25	5.6	
Anal	0	0.0	13	2.9	
Multiple genital injuries	0	0.0	20	4.4	
* Missing values*	*6*		*59*		
**Extragenital injury**					0.735
None	9	36.0	102	23.7	
Head, neck, or trunk	1	4.0	29	6.7	
Upper limb	1	4.0	17	4.0	
Lower limb	3	12.0	61	14.2	
Multiple extragenital injuries	11	44.0	221	51.4	
* Missing values*	*20*		*77*		
**Alcohol use**					0.023
No	19	57.6	162	37.5	
Yes	14	42.4	270	62.5	
* Missing values*	*12*		*75*		
**Drug use**					0.014
No	24	77.4	386	91.0	
Yes	7	22.6	38	9.0	
* Missing values*	*14*		*83*		

*n* quantity, *%* percentage, *SD* standard deviation

^a^ March 12 to May 11, 2020, October 23, 2020 to February 28, 2021

### Victim characterization

There was no difference in age of the patients presenting to the emergency department during the first year of the pandemic compared to the evaluated pre-pandemic period (COVID-19: mean [SD] = 26.6 [10.6] vs. pre-pandemic: mean [SD] = 26.6 [12.0]). The majority of the victims claimed to have had a relationship with their offender during and before the pandemic (COVID-19: 62.3% [n=66/130] vs. pre-pandemic: 54.3% [n=506/1037], p=0.133, Table 1]).

During the lockdown, the offender was more often an acquaintance of the victim compared with the corresponding pre-pandemic years (72.2% [n=26/45] vs. 56.4% [n=254/507], p=0.065, Table 2).

### Site of the offense

There was no difference in the location of the offense during and pre-pandemic (p=0.287, Table 1). In the majority of the cases, the sexual assault took place in the apartment of the offender (COVID-19: 36.3% [n=40/130] vs. pre-pandemic: 31.1% [n=100/1037]) followed by the apartment of the victim (COVID-19: 26.4% [n=29/130] vs. pre-pandemic 25.6% [n=82/1037]). A nightclub/bar was less often the place of sexual assault during COVID-19 (10.9% (n=12/130] vs. 18.7% [n=60/1037]).

During the lockdown and corresponding pre-pandemic periods, most assaults occurred in the home of the offender (36.8 [n=14/45] vs. 33.0% [n=58/507]) or the home of the victim (28.9 [n=11/45] vs. 27.8% [n=49/507]). During the lockdown a bar/ nightclub was less often the site of the offense (7.3% [n=3/45] vs. 19.9% [n=34/507], p=0.319, Table 2).

### Criminal report

More than 90% of the women, both during and before the pandemic, presented to the emergency room after a first-time sexual assault (Tables 1 and 2). During the pandemic however, only 49.5% (n=54/130) of all patients reported the sexual assault to the police, whereas 73.9% (n= 599/1037) of patients filed a police report in the pre-pandemic period (p<0.001, Table 1). Similar findings could be shown during the lockdown. Only 50.0% (n=19/45) of the victims filed a police report during the lockdown compared to 76.5% (n=309/507) in the corresponding pre-pandemic periods (p<0.001, Table 2).

### Injury pattern

During the first year of the pandemic, the gynecology examination revealed fewer genital injuries (14.2% [n=15/130] of affected individuals of sexual assault) in comparison to 25.2% [n=236/1037]) during the previous years (p=0.020). There was no difference in findings of extragenital injury, detected by the forensic medical examination (COVID-19: 76.6% [n=59/130] vs. pre-pandemic: 76.7% [n=68571037], p=0.871, Table 1). There was also no difference in genital or extragenital injury patterns during the lockdown, compared to the previous years (Table 2).

### Substance use

More patients declared voluntary use of illegal substances during the first year of the pandemic (COVID-19: 19.5% [n=16/130] vs. pre-pandemic: 9.3% [n=82/1037], p=0.003, Table 1).

During the lockdown, fewer affected individuals reported a voluntary consumption of alcohol in the context of the sexual assault (42.4% [n=14/45] vs. 62.5% [n=270/507], p=0.023), whereas the voluntary use of illegal substances increased during the periods of government-imposed restrictions (lockdown: 22.6% [n=7/45] vs. corresponding pre-pandemic: 9% [n=38/507], p=0.014, Table 2).

## Discussion

Only during strict contact restrictions and stay-at-home orders did the number of sexual assault vicitims presenting to an emergency department decline in comparison to previous years. The overall number of sexual assault cases presenting to an emergency room in a large metropolitan area did not change during the COVID-19 pandemic in comparison to the previous years.

These findings are in line with results from Eastern Denmark, which indicate that the pandemic did not impact the number of examinations conducted after rape [19]. This is in contrast with data from India, which confirmed an association between contact restrictions and a reduction of sexual offenses [15]. In all given analyses, the authors were only able to use the information of women seeking medical care in an emergency department. This cohort most likely reflects a small proportion of women experiencing sexual offenses.

### Decrease of reporting rates

Our findings, however, indicate that the pandemic had an impact on the reporting rates of sexual assaults. Fewer affected women claimed to have filed a police report during the pandemic compared to previous years. In addition, fewer women claimed to have filed a police report during the lockdown, as compared to the corresponding times of previous years. This finding is in line with the results of a study from England, also showing a decrease in rape and serious sexual offense reporting during the COVID-19 pandemic and especially during the lockdown [20]. It could be speculated that the policy of mandatory stay-at-home orders and the closure of clubs and bars reduced the opportunities for rape and sexual assault. On the other hand, an increase in reported domestic violence in response to stay-at-home orders during the COVID-19 pandemic, has been reported [16, 19, 21]. Friis-Rødel et al. showed that victims were more likely to report the assault if the perpetrator was a stranger [22]. Our data did not show a difference in the victim-offender relationship before and during the pandemic. The group of victims assaulted by an acquainted offender could however be underrepresented if these victims are afraid to seek medical care after the offense. Reasons for not reporting sexual assault or rape in times of crises like the COVID-19 pandemic could also be the fear or shame of troubling authority with a personal issue or less availability of services [23]. A possible further explanation might also be that victims were reluctant in reporting sexual offenses when they happened in illegal venues.

### Decrease in genital injuries

Interestingly, fewer genital injuries were reported during the COVID-19 pandemic. The forensic relevance of genital injuries, in general, is difficult to assess because the occurrence of genital injuries after sexual assault varies from 5 to 87% in the literature [24] and neither the existence nor the absence of these injuries rule out the lack of consent [25]. Factors thought to be associated with genital injuries after sexual assault are controversial [25], but the forensic evidence from these injuries is established to evaluate an alleged penetration mechanism and the details of the offense that have been previously reported [8]. It has been shown that a lower occurrence of genital injuries in association with the administration of sedatives [8, 26] as intoxicated and sleepy victims presented less resistance and might therefore be at a lower risk of genital injuries [27]. Accordingly, we have demonstrated an increase in illegal drug use (rather than alcohol) by victims during the COVID-19 pandemic and the lockdown periods.

### Increase of illegal substance use

During lockdown periods, fewer victims reported consumption of alcohol in association with the sexual offense, if anamnestic data are reliable. It could be assumed that gatherings and parties happened illegally, with more readiness for the consumption of illegal substances. Additionally, the shutdown of restaurants, bars, and clubs offered fewer opportunities for social alcohol consumption, which could explain less consumption of alcohol during the lockdown periods. Social isolation, stress, economic burden, and limited access to medical treatments have been correlated with a general increase in illegal drug use during the COVID-19 pandemic [28].

Despite home confinement during lockdown periods, our data did not show a difference in crime sites. Before the pandemic and during the pandemic the site of the offense was mainly the private home of the suspect or victim, followed by a nightclub/bar. This is in line with the data from the largest study of sexual assault cases from Fryszer et al. [9] indicating that 2/3 of all sexual offenses happen in private rooms. However, even during the lockdown periods, when nightclubs and bars were officially closed, these public places were still reported as offense sites. It can be assumed that nightlife still happened illegally.

### Limitations of the study

It is important to acknowledge that our data were collected retrospectively through the evaluation of individual patient charts. Some information, especially about the victim-offender relationship might be missing, if the patient did not feel the need to report this information. Additionally, information about the consumption of alcohol or illegal substances was only obtained by anamnestic data and not verified by laboratory results. Furthermore, our data was only obtained from one emergency room of a university medical center in a large city in Germany and results might be different in more rural areas.

## Conclusions

The COVID-19 pandemic has offered a unique opportunity to examine the influence of crisis and contact restrictions on patterns of sexual assault victims within a German metropolitan city. To our knowledge, this study is the very first to demonstrate a decrease in female-reported sexual assault to an emergency department of a university medical center in Germany during the pandemic. The decrease in reporting rape and sexual offenses with a constant number of cases is alarming and emphasizes the importance of providing support for sexual assault victims, especially in times of crisis. Fear, shame, and lesser availability of services [23] may offer initial explanations, but further research is needed to evaluate the reasons for decreased reporting rates. The increase in illicit drug usage by sexual assault victims during the pandemic also highlights the need for preventive measures in the future.

## Key points

1. The overall number of sexual assault cases did not decrease throughout the pandemic, but only during the periods of strict contact restrictions fewer cases of sexual assault were reported.
2. Fewer victims reported sexual assaults to the police during the pandemic.
3. Most assaults occurred in the home of the offender.
4. Although the consumption of alcohol decreased during the lockdown, illegal substance usage increased.

## Data Availability

Data available on request from the authors.
